# How to Join a Wave: Decision-Making Processes in Shimmering Behavior of Giant Honeybees (*Apis dorsata*)

**DOI:** 10.1371/journal.pone.0036736

**Published:** 2012-05-08

**Authors:** Gerald Kastberger, Frank Weihmann, Thomas Hoetzl, Sara E. Weiss, Michael Maurer, Ilse Kranner

**Affiliations:** 1 Institute of Zoology, University of Graz, Graz, Austria; 2 Institute for Computer Graphics and Vision, Graz University of Technology, Graz, Austria; 3 Institute of Botany, University of Innsbruck, Innsbruck, Austria; University of Arizona, United States of America

## Abstract

Shimmering is a collective defence behaviour in Giant honeybees (*Apis dorsata*) whereby individual bees flip their abdomen upwards, producing Mexican wave-like patterns on the nest surface. *Bucket bridging* has been used to explain the spread of information in a chain of members including three testable concepts: first, *linearity* assumes that individual “agent bees” that participate in the wave will be affected preferentially from the side of wave origin. The *directed-trigger* hypothesis addresses the coincidence of the individual property of trigger direction with the collective property of wave direction. Second, *continuity* describes the transfer of information without being stopped, delayed or re-routed. The *active-neighbours* hypothesis assumes coincidence between the direction of the majority of shimmering-active neighbours and the trigger direction of the agents. Third, the *graduality* hypothesis refers to the interaction between an agent and her active neighbours, assuming a proportional relationship in the strength of abdomen flipping of the agent and her previously active neighbours. Shimmering waves provoked by dummy wasps were recorded with high-resolution video cameras. Individual bees were identified by 3D-image analysis, and their strength of abdominal flipping was assessed by pixel-based luminance changes in sequential frames. For each agent, the directedness of wave propagation was based on wave direction, trigger direction, and the direction of the majority of shimmering-active neighbours. The data supported the *bucket bridging* hypothesis, but only for a small proportion of agents: linearity was confirmed for 2.5%, continuity for 11.3% and graduality for 0.4% of surface bees (but in 2.6% of those agents with high wave-strength levels). The complimentary part of 90% of surface bees did not conform to *bucket bridging*. This fuzziness is discussed in terms of self-organisation and evolutionary adaptedness in Giant honeybee colonies to respond to rapidly changing threats such as predatory wasps scanning in front of the nest.

## Introduction

*Shimmering* behaviour in Giant honeybees (*Apis dorsata*) [1], [2] involves a display of social waves with antipredatory impact [3]–[9]. These waves originate at discrete areas on the surface of the nests, where *generator bees* have been identified [10] as leaders in the general responsiveness to external cues. These generator bees raise their abdomen first and affect their nest mates around them to follow them in sequential order. Such behavioural cascades generate self-organized [11]–[13] patterns propagating across the nest surface in a fraction of a second. The visual domain of these spatial time patterns [movies S1, S2, and S3] aim at repelling external addressees such as predatory wasps [7], [14], [15] or mammals. It is reasonable to assume that the mechanoceptive domain [16] of shimmering is important for colony-intrinsic communication [16], because the waves affect most of the colony members not only in the surface layer, but in all layers of the bee curtain [7], [14]; they are even supposed to influence the bee curtain on the non-shimmering, opposite side of the comb [17].

**Figure 1:**
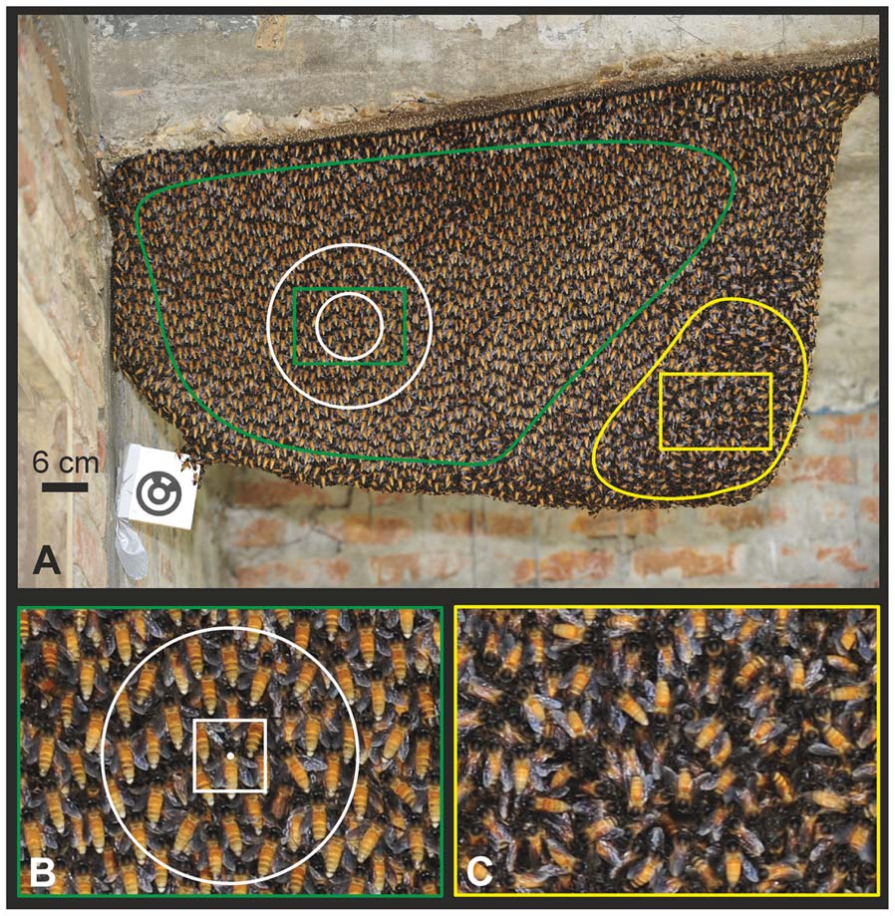
Experimental Giant honeybee nest. Chitwan (Nepal) in January 2009. (A) View of the whole nest; the green line gives the quiescent zone, where the bees are positioned with their heads upwards and the abdomens downwards; outside of this zone are the rim areas (left side and bottom), the attachment zone (top), and the mouth zone (right) marked with the yellow line; the green and yellow rectangles show nest areas which are displayed at a bigger scale in (B, quiescent area) and (C, mouth zone); white circles show the *near neighbourhood* (r<40 mm) and the *far neighbourhood* (r<100 mm) of the selected *focus bee*, which is positioned in the centre of the circles. The outer black circle within the white size marker (bottom left of the nest) is 6 cm in diameter. (B) Area of the quiescent zone; the white dot shows the position of the thorax of the selected *focus bee*, defining the centre of the white open circle (r = 40 mm) of the *near neighbourhood* and of the white square which indicates the detection area (60 x 60 px) of the selected bee to assess the _rel_XYmov values (see Methods). Note the differences in orientation of the surface bees between quiescent zone (B) and mouth zone (C).

The specific involvement of surface bees in wave propagation is not well understood. However, several features of shimmering can be compared with wave-like processes in general. For instance, colliding wave-fronts which extinguish each other would suggest the presence of refractory processes, which have been extensively studied in excitation physiology (e.g. [17]). Second, similar to water or sound waves [18], [19], shimmering waves reorient or fade out (Fig. 1; movies S1, S2, and S3) at physical hindrances to the propagation of waves. In Giant honeybee nests, such hindrances can be architectural or functional structures, which are given at rim areas, attachment zones or at the *mouth* zone (Fig. 1C; [7], [20]). Third, similarity exists with other types of social waves such as the Mexican waves of human spectators in football stadiums, which has led some authors to classify shimmering as a Mexican-wave-like process [16], [21].

At first sight, and to the naked eye, shimmering appears to spread mostly in *linear* tracks. If examined more closely, the waves reveal a series of group-level properties [11]. An example for the higher complexity in shimmering is the *saltatoric* spreading pattern [14], [22], where information “jumps” from one group of bees to a neighbouring one. Another indication for complex group-level properties is the variability of the directedness of propagation, which regularly shows linear, curved or even spiral patterns [23], [24] (movies S1).

**Figure 2:**
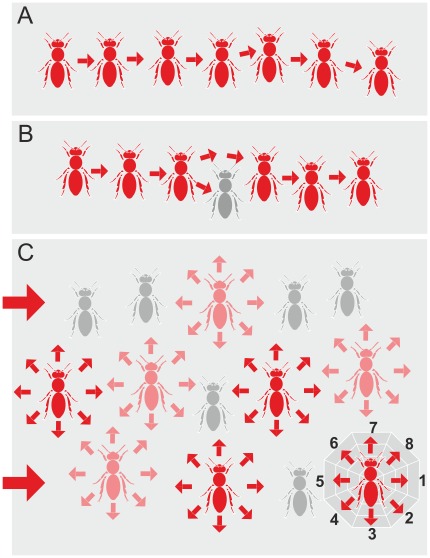
Schematic of *bucket bridging* during shimmering and the aspects of *linearity* and *continuity* in propagation. (A) *Linear* and *continuous* transmittance: Information is transferred (symbolized by red arrows) from one agent bee to the next member of the chain. (B) *Linear* but *discontinuous* transmittance: one agent bee in the chain fails to act as transmitter but information is by-passed to the next agent in the chain. (C) Schema of a sample wave which spreads from left to right (dir_WAV_ = _from_L_to_R) explaining the complex situation of the bee curtain of *A. dorsata* during shimmering, with *non-linearity* and *discontinuity* of transmittance; three types of agent bees are noted: *red*, strong transmittance corresponding to wave strength levels of c_ws_>2; *light red*, weak transmittance corresponding to wave strength levels of 1≥c_ws_≤2; *grey*, no transmittance corresponding to a wave strength level of c_ws_ = 0 (these bees were detected as agent bees but not further considered in the evaluation). The arrows signify that information is transmitted from any abdomen-flipping agent bee into all eight neighbourhood sectors (for definition of dir _Nh_, see sketch at the bottom right, see Table 1 for definitions of angular sectors).

In this study, we investigated the propagation mechanisms of shimmering, paying particular attention to the principle of *bucket bridging* [14]. *Bucket bridging* can be regarded as the simplest group-level property in wave propagation, whereby cues are passed on from one individual in a chain to another (Fig. 2A). The term *bucket bridging* originates from a collective human behaviour, where a bucket of water is delivered to the site of fire by a chain of humans who pass the bucket from one individual to another. If one member in the chain quits his duty, the bucket transfer will be delayed, stopped (Fig. 2B), or rerouted to other individuals of the same or of another chain (Fig. 2C). If the propagation of shimmering waves was to conform strictly to the *bucket-bridging* hypothesis, the wave would spread according to the principles here defined as *linearity, continuity* and *graduality.*

*Linearity* in *bucket bridging* assumes that information is delivered sequentially from one member of a chain to the next. In the case of shimmering, *linear* propagation would result in the wave front proceeding steadily from one surface bee to the next. An individual bee, here termed *agent bee*, with a disposition to participate in the wave, will be affected by the wave front preferentially from the site of wave origin. The *directed-trigger* hypothesis addresses here the coincidence of the individual property of *trigger direction* with the collective property of the *wave direction*. *Continuity* in shimmering is defined as the transfer of information by groups of surface bees without being stopped, delayed or re-routed. The stimuli that trigger continuous shimmering would come from the direction of maximal activity of neighbouring agents. This shimmering principle will be addressed by the *active-neighbours* hypothesis, investigating the coincidence between the angular sector where the majority of active neighbours are positioned, and the *trigger direction* of the selected individual. *Graduality* is the third principle of *bucket bridging* and also refers to the interaction between a surface bee and her neighbours. The *graduality* hypothesis assumes a proportional relationship in the strength of abdomen flipping of an agent bee and her previously active neighbours.

However, shimmering depends upon a series of factors that can considerably curtail the above-mentioned principles of *bucket bridging*. Such factors include the architecture and the partitioning of the nest (Fig. 1A; [14]), the defence state of the colony [10], and the sensory equipment of the bees. Individual surface bees are believed to be free to decide to join or not to join a shimmering wave [14], [16]. If they do, they flip their abdomen with a variable angle of up to 120°, synchronizing and cascading their behaviours collectively, which, for the external observer, results in a wave-like pattern [14], [25], [26] with a high level of complexity, sophistication and thus, unpredictability. Considering these restricting factors it seems likely that wave propagation in shimmering does not obey the strict laws of *bucket bridging* alone.

This paper addresses the applicability and restrictions of the *bucket-bridging* hypothesis for wave propagation in shimmering [7] in Giant honeybees, focusing on the principles of *linearity, continuity* and *graduality*. We precisely determined the position of individual agent bees over time in the three dimensions of space, thereby gaining information regarding the trigger conditions and the neighbourhood of *focus bees*. The behaviours were recorded with high-resolution and high-speed video. Bees at the surface of the bee curtain were re-identified on an individual basis [16] frame by frame by stereo imaging and automated tracking, enabling us to analyse the underlying mechanisms of wave propagation.

## Materials and Methods

### Ethics Statement

The office of the rector of the centre for international relations of Tribhuvan University (Kathmandu, Nepal) certified the research expedition entitled “Study on the behaviour of the Giant honeybees: Observations and Recording of behaviours at the nesting site” in Chitwan district of Nepal.

### Experimental Site and Recording

**Figure 3:**
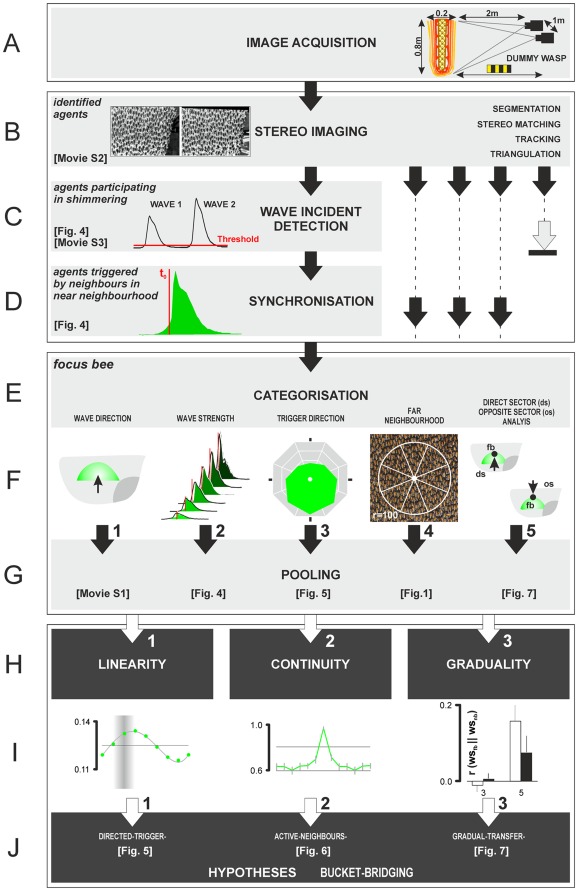
Schematic of the evaluation process to analyse properties of the propagation process in shimmering. The light-grey flow charts (A–G) address the single-agent analysis from image acquisition, stereoscopic imaging of individuals, wave incident detection, to the synchronization, categorization, and pooling of wave incidents. The black arrows and dashed lines on the right side symbolize that the stereoscopic analysis produced further data for hundreds of agent bees simultaneously. (A) The experimental nest was captured by two frame-locked video cameras positioned at an angle of 30° two meters in front of the nest. A single recording session lasted 15 s and included up to two shimmering waves which spread across the nest surface. Shimmering was elicited by a dummy wasp. (B) In the offline data assessment phase the acquired images were processed as follows: *Segmentation* distinguished single agent bees in the densely packed clusters of bees on the surface of the nest in each of the paired images, *stereo matching* identified corresponding agent bees in both paired images. These two processes enabled *stereo tracking* of the agents in subsequent frames throughout whole film scenes and the *triangulation* of their thoracic positions regarding the three dimensions of space (x,y,z) [14]. (C) The stereoscopic analysis delivered identities of agent bees in subsequent image sequences. The arrival of the wave front at an individual agent was recognized by a movement detection algorithm (see Fig.1B, white square). The grey arrow on the right with the stop bar symbolizes that a minority of identified agents did not participate in shimmering and was not evaluated further. (D) The detection of active participation in the wave allowed synchronization of wave incidents of individual agent bees at different positions of the nest. (E,F) Four categorizations of participating agents were discerned to define single *focus bees*: the wave direction dir_WAV_ (F_1_); the wave strength level (c_WS_) as a measure of the response strength, which refers to the maximal _rel_XYmov values of a wave incident (F_2_); the trigger direction dir_TRIG_ gives the angle of the triggering neighbour in the *near neighbourhood* (F_3_); the oval area with its displacement from the centre shows the direction of the maximum activity of neighbours in the *far neighbourhood* (F_4_); in (F_5_), the paradigm of *direct-sector* and *opposite-sector* analysis was introduced. (G) Pooling of synchronized and categorized 3D data (Δx, Δy, Δz) and wave strength values _rel_XYmov. (H-J) Using the parameters defined in F_1–5_, three criteria of *bucket bridging* were assessed (dark-grey flow charts H,I: *linearity*, *continuity*, *graduality*) and the respective hypotheses (*directed-trigger-*, *active-neighbours-* and *gradual-transfer-*) were tested (J_1–3_). The panels in D, F and I illustrate the results of the processes given in the grey or black boxes (see referenced figures and movies for details).

The shimmering behaviour of Giant honeybees (*Apis dorsata*) was studied under field conditions in Chitwan, Nepal, during two expeditions in February 2009 and November 2010. To obtain a detailed view of the movements of individual bees within the entirety of the bee curtain [7], [14] the motions of hundreds of surface bees were measured simultaneously in the three directions of space. This was achieved by an adaptation of the stereoscopic imaging principle [27] with its fundamental algorithms [16]: segmentation [28], matching [29], [30] and reconstruction [31], [32] by tracking and triangulation (Fig. 3A,B). Two video cameras (type DALSA Falcon 4M60) delivered black-and-white images with a resolution of 2352×1728 pixels (px). From the given working distance of 2 m, and with a calibrated focal length of 53 mm, about two thirds (700 mm in diameter) of the nest were recorded, whereby one px represented ∼ 0.30 mm in metric real-world coordinates. Therefore, the characteristic abdomen width of 6 mm of a bee was imaged by roughly 20 px. The cameras were able to capture 60 frames per sec (fps) and so resolve the abdomen-flipping phase of an individual bee of 200 ms within 12 frames (for further details see [16]).

### Identification of Surface Bees

In a Giant honeybee nest, the colony members are arranged at both sides of the central comb in several layers. A nest has several functional regions (Fig. 1) such as the *mouth* zone, the attachment zone, the rim zone and the quiescent zone [14], [20], [33], [34]. Shimmering behaviour is displayed primarily by the surface bees in the quiescent zone [7], [35]. More than 600 individually identified surface bees per frame were tracked (Fig. 3A,B; the identification of the positional coordinates defines them as “*agent bees*”), summing up to 14549 *wave incidents* which were documented in successive film sessions (defined as a sequence of images tracked continuously in the course of a single experiment). The identification of individual agent bees throughout a single session is challenging because Giant honeybees in a colony are extremely similar in morphology, are densely clustered, and show rapid movements in 3D during their abdominal flipping [16]. In addition, an individual bee that has sensed an incoming wave front due to the 3D movements of her neighbours is free to decide whether or not to join the wave, and if she joins, whether to raise her abdomen strongly or weakly. Weak participation of individuals in shimmering is difficult to detect by automated analysis. Furthermore, it is critical to distinguish active “movements”, i.e. abdominal flipping, from passive “motions” caused by the surrounding bee curtain.

### Definition of the Wave Directions

The architecture of the bee curtain of Giant honeybee nests is determined by three characteristics: first, by the orientation of surface bees which hang, particularly in the *quiescent* areas, with their heads upwards and their abdomens downwards (Fig. 1B; [7], [14]); second, by the particular structure of the nest, which suspends freely from overhead structures to which it is attached; and third, by the polarity between the nest centre of the mouth zone and the quiescent periphery. Four key directions of wave spreading were defined (dir_WAV_ = 1-4; Table 1), namely two horizontal directions (*from Right to Left* [_from_R_to_L], *from Left to Right* [_from_L_to_R]) and two vertical directions (from *Bottom to Top* [_from_B_to_T], *from Top to Bottom* [_from_T_to_B]), numbered clockwise, starting with _from_R_to_L. For that, shimmering waves in these key directions were identified visually from recorded movies.

**Table 1:** Definitions of categories of directions (*dir*) and sectors around a *focus bee*.

Directions	Sectors		
from whence the activities started	CATEGORIES dir_Nh,_ dir_TRIG_	CATEGORIES dir_WAV_	ANGULAR RANGES α_Nh,_ α_tb_, α_TRIG,_ α_WAV_
*from Right*	1	1 [_from_R_to_L]	0±22.5°
*from Bottom-right*	2		45±22.5°
*from Bottom*	3	2 [_from_B_to_T]	90±22.5°
*from Bottom-left*	4		135±22.5°
*from Left*	5	3 [_from_L_to_R]	180±22.5°
*from Left-up*	6		225±22.5°
*from Top*	7	4 [_from_T_to_B]	270±22.5°
*from Right-up*	8		315±22.5°

α_Nh_±22.5°, the sector of neighbourhood with α_Nh_ as its central direction; α_TRIG_−22.5°<α_tb_ <α_TRIG_+22.5°, the angular range where the triggering bee is located, synonymous to the trigger angle; α_WAV,_ the angle from where the wave is spreading.

### Assessment of the Motion Strength at the Individual Level

**Figure 4:**
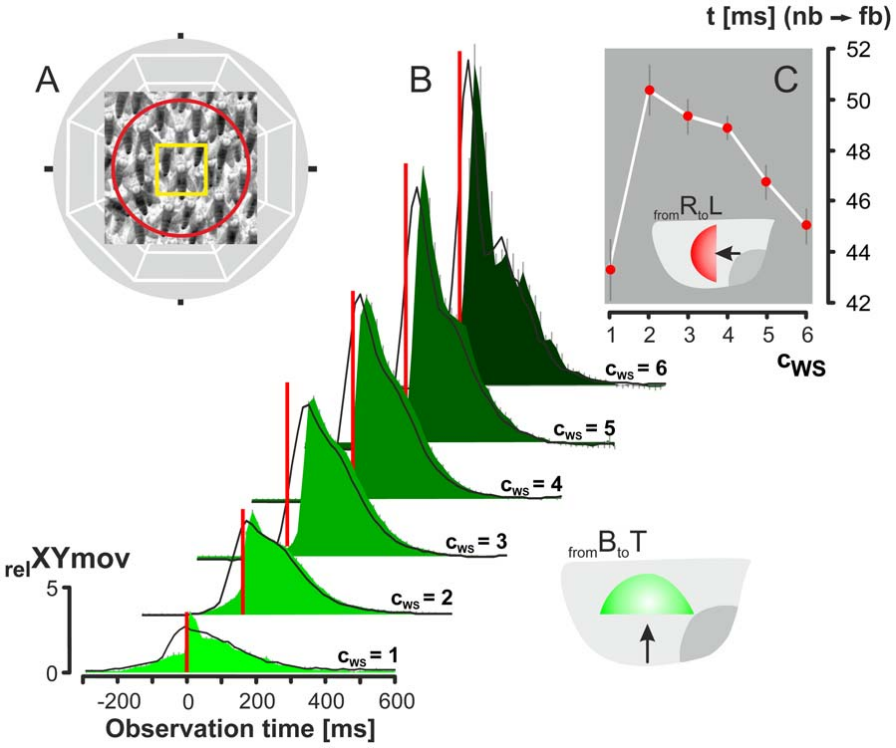
The time courses of wave strength of *focus bees* and their triggering neighbours. (A) Details about the assessment of the image-based _rel_XYmov values (see Methods); yellow square gives the 60×60 px region of interest (ROI) around the selected *focus bee*; the red circle defines the *near neighbourhood* (r < 40 mm); the white lines in the background show the eight sectors of neighbourhood (dir_Nh_ = 1–8). (B) Time courses of the _rel_XYmov values of *focus bees* at different wave strength levels (dir_WAV_ = _from_B_to_T; c_ws_ = 1-6; n = 2908 wave incidents). The contours of the green areas show arithmetic means, vertical grey bars show mean errors. Black lines are means of _rel_XYmov values of the triggering neighbours (radius_Nh_<40 m) regarding the paired, *focus bee*-related wave incidents; mean errors are not displayed here. Red vertical bars signify the time points t_0_ defined by the rapid onset of shimmering activity (quantified by the ordinate_ rel_XYmov values) of the *focus bees*. (C) How long does it take that information of shimmering is transferred from the neighbours in the *far neighbourhood* (radius_Nh_<100 mm) to the *focus bees*? Estimation exemplified for dir_WAV_ = _from_R_to_L; abscissa, wave strength level; ordinate, time interval in ms in which information has been transferred, with means (red dots) ± SE (black vertical bars), n = 14 threshold levels (_rel_XYmov = 1.0–2.3 in steps of 0.1) for the neighbourhood activity; ordinate values are calculated by weighted interpolation and cross correlation of the time courses between *focus bees* (*fb*) and their trigger neighbours (*nb*) of the same wave strength level (N_fb_ = 2824; N_nb_ = 29237).

For each identified agent bee (Fig. 3A,B) a set of data was available frame by frame [16]. We introduced the parameter _rel_XYmov (Fig. 3C,D) to categorize the motion strength of individual agents throughout the experiment [16]. This measure allowed the assessment of the precise time of the arrival of a wave at an agent’s position. A reliable trigger criterion was found by detecting the luminance changes in two sequential frames (f_i-1_, f_i_) in a pixel-wise subtraction creating a difference image [16]. A *region of interest* (ROI; Fig. 1B, Fig.4A) of 60×60 px around an agent bee was defined, with the centre point positioned in the middle of the thorax corresponding to 18×18 mm in real-world coordinates. The size of the ROI was chosen in conformance with the mean side-to-side distances between surface bees [16] covering 80% of the agent bee, including most of the abdomen, and also considering the stretched-wing condition where the two wings on each side are presented separately without overlapping, which is characteristic of surface bees. A larger sensor field would have interfered too much with the motion activities of the neighbours. We recorded 8-bit px values regarding the differences in luminance (Δ lum) between two successive frames assessed by pixel-operated subtraction [16] with the references as *black* [Δ lum = 0] and *white* [Δ lum = 255].

These Δ lum values represented motion activities of a selected agent bee regarding the whole body within 16.67 ms. These motions were quantified by the parameter _rel_XYmov and include positional changes in horizontal (*x*-) and vertical (*y*-) directions, but also movements of head, abdomen, and extremities, such as legs, antennae or wings. This value is therefore affected by various behavioural contexts: first, by passive deflections of the whole body in x-, y- and z-directions which originate from the immediate neighbourhood [16]; second, by active movements of the body parts such as observed in flickering [36] or shimmering [14], and third, by locomotor activities of the whole body such as moving around on the nest surface or penetrating into deeper layers of the nest. For quiescent conditions the Δ lum values typically were on average 3 per px, which summed up to _rel_XYmov = 1.08 * 10^4^ for the whole 3600 px of the ROI. Massive shimmering activity reached on average tenfold _rel_XYmov values. Therefore, the _rel_XYmov value was an appropriate parameter to quantitatively describe the level of the individual participation of an agent bee in shimmering waves, i.e. the *wave strength* (*ws*) of a given wave incident [16].

### Determination of the Time Zero of a Singular Wave Incident

When a wave front reaches an agent bee, weak passive deflections are detectable, but when an agent bee starts raising her abdomen the *ws*-value sharply rise and peak within 40 ms (Fig. 3C). This effect was utilized, first, to determine the starting time (t_0_) of an individual shimmering *incident*. The time point zero *t_0_* was defined one frame before the time *t_1_* at which the sharp rise of _rel_XYmov value was detected (t_0_ = t [f_1_–1]). Second, this rapid rise of the _rel_XYmov value was also utilized to synchronize the multitude of shimmering incidents which took place, simultaneously or successively, at the various agents of the whole nest. For that, we defined an additional threshold level of Δ ws_th_ = 10 to identify an agent bee which participated in the shimmering wave. An agent must have shown Δ ws-values which exceeded this threshold for at least five successive frames (Δ _rel_XYmov (t_1_,…t_5_) > Δ ws_th_), to exclude noise-triggered processes. Both rules were important because the parameter _rel_XYmov was also used to categorize eight levels of participation of agent bees in shimmering (c_ws_ = 1–8). The assignment of c_ws_ = 1 served as the minimum level of shimmering activity of an agent bee (Fig. 3C,D) and unambiguously excludes sub-threshold shimmering activities.

### The Concept of the *Focus Bee*

The participation in shimmering varied strongly from agent to agent. This individual bias is caused by a series of agent-specific factors such as the location in the nest, the individual’s age, the direction of wave spreading at the agent’s location, the level of the passive participation (i.e. motion without abdominal flipping) or of the active participation (i.e. motion caused by abdominal flipping at variable strength), and lastly the participation of the neighbours of the selected agent in the wave. Therefore, we introduced the concept of the *focus bee* (*fb*) to enable the pooling and statistical evaluation of the data in particular. For every *focus bee*, we defined two areas of neighbourhood (Fig. 1A,B), the *near neighbourhood* (radius_Nh_<40 mm) and the *far neighbourhood* (radius_Nh_<100 mm), and selected those neighbour bees (*nb*) which participated in shimmering before the wave front had arrived at the *focus bee*. As a consequence, an agent bee was treated in the evaluation either as *focus bee* or as neighbour bee of a previously defined *focus bee*.

### Triggering the Participation in Shimmering

For a given *focus bee*, the _rel_XYmov value (Fig. 3C,D) was utilized to define the arrival time (t_0_) of the shimmering front and also the level of participation in shimmering (c_ws_-value). For the nest mates around a given *focus bee*, the rapid rise of the _rel_XYmov value at t_1_ = t[f_1_] was used to assign the potential trigger neighbour (*tn*) for a given shimmering *incident* by considering three criteria: (a) The trigger neighbour had already participated in the same wave, but only for a maximum of 5 frames (Δt≤88 ms) before the *focus bee* itself had started her shimmering activity. (b) The trigger neighbour was closest of all other candidates to the *focus bee* and was positioned in her *near neighbourhood* which made up not more than 5–7 agents. This rule excluded those bees from analysis which generated daughter waves [22]. Such surface bees were flipping their abdomens singularly or in a small group and were farther away from other active bees than defined by the *near neighbourhood*. (c) Furthermore, the angular position of the trigger neighbour (α_tn_) defined the *trigger direction* (dir_TRIG_) of the *focus bee*. For that, eight sectors of neighbourhood (dir_Nh_ = 1–8) circularly around the *focus bee* were defined in steps of angular ranges Δα_Nh_ = 45° (Table 1). Consequently, the *trigger direction* of the *focus bee* was defined by the angular sector (dir_TRIG_ = 1–8) in which the trigger neighbour was positioned (Table 1).

### Relative Time Scales

The time point t_0_ (Fig. 3D) of a *focus bee* defined the frame before the *focus bee* displayed the supra-threshold movement of abdomen flipping. This reference established a time scale for both partners in the trigger process, the triggered *focus bee* and her triggering neighbour. The time courses of the movements of both agents (*fb,tn*) were registered and synchronized to the time zero of either the *focus bee* herself (t_0_[*fb*]) or in the case of the triggering neighbours (*tn*), to the time zero of the *focus bee* to which they had been referred to. This synchronization conjoined the movements of both agents by the positional (*x,y,z*) parameters and by the wave strength (_rel_XYmov). Such wave processes of identified *focus bees* and of their neighbours collected at different locations and times were consecutively synchronized and sorted according to behavioural categories such as wave strength c_ws_, main wave direction dir_WAV_ and trigger direction dir_TRIG_ (Table 1).

### Stimulation by Dummy Wasps

Waves were elicited using a cable car device [16] with a dummy wasp positioned at the outer sun-oriented nest side below the mouth zone (shown on the bottom right side of the experimental nest in movies S1, S2, and S3). Using this computer-controlled device [16], [35], the dummy wasp could be moved at variable velocities (0.1 - 0.5 m/s) and directions (Fig.3A). The dummy consisted of Styrofoam (L×W×H: 40×15×15 mm) with white, yellow and black painted stripes. The dummy was fastened to the cable car by a thin thread, so that is was freely swinging with the length axis horizontally. In the experiments described here, the dummy wasp was moved at an angle of 90° to the nest surface. For more intense stimulation we used the manual technique of moving a rod with a swinging dummy of the same geometrical pattern as under cable-car conditions.

## Results

### Categorization of Active Participation in Shimmering

Individual agent bees were identified on the surface of a Giant honeybee nest by stereoscopic imaging (Fig. 3A,B; [11]) for 66 manually selected shimmering waves (Fig. 3C). Twenty-two independent data sets of four wave directions (dir_WAV_ = 1–4) were analysed. Only a subset of 53.06±3.05% of identified agent bees were found to participate actively in shimmering, defined by flipping their abdomens at an above-threshold strength level (c_WS_ = 1-6). Bees that did not participate above this threshold were excluded from further analyses. *Focus bees* (Fig. 3E) received the mechanical cues from their nest mates in the *near neighbourhood*. The category of *focus bees* constituted 75.72±2.90% (n = 22 data sets of 10715 identified bees) of those bees which actively contributed to shimmering. The complementary part of 24.28% active bees flipped their abdomens without having received a mechanical cue from their immediate neighbours. These bees were likely to be members of an alternative spreading mechanism [22] and were excluded from further evaluation (Fig. 3E–H).

### The Time Courses of Wave Incidents of *Focus Bees* and Their Triggering Neighbours

By definition, the *focus bees* started their participation in shimmering at t_0_, after they had received triggering cues from their active neighbours. Prior to t_0,_ the shimmering activities rose slowly within 200 ms (Fig. 4B) which was caused in the arrival phase of the shimmering front by the movements of the neighbours [16]). This passive motion of the *focus bees* can be considered as a potential decision-enabling cue which may trigger, or at least influence, the start and strength of the subsequent participation. In particular at higher wave strength levels, the subsequent course of _rel_XYmov values displayed damped oscillations, which were due to resonance conditions of the bee curtain [16].

Fig. 4B also shows in black lines the curves of the neighbours in the *far neighbourhood* of the *focus bees* assembled in their trigger direction. For that, only those neighbours were selected that had flipped their abdomens at the same wave strength as the *focus bee*. This enabled the assessment of the time for the information to spread from the neighbours to the *focus bees* which lie at 47.29±1.12 ms (Fig. 4C; mean ± SE; n = 6 wave strength levels; averaged for threshold levels of 1.0≥_rel_XYmov≥2.3, Fig. 3C). For small and higher wave strength values (c_WS_ = 1; c_WS_>4) the transfer times are significantly shorter. Taken in account the average distance of 62 mm between the *focus bee* and neighbours in the *far neighbourhood*, *bucket bridging* achieved a speed of 1.311±0.030 m/s for this step of wave propagation.

### Testing *Linearity* of Wave Propagation

In shimmering, the *directed-trigger* hypothesis.



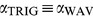
(1a)can be tested at each *focus bee* by the coincidence of *trigger direction* and *wave direction* (Fig. 3H_1_). We proved this aspect of *linearity* (Fig. 3I_1_) using four independent data sets (dir_WAV_ = 1–4; Table 1) of selected sample waves. In detail, we checked whether and how the angular positions of the triggering neighbour coincided either with the direction from where the wave came (conforming to equation 1a, ) or with the opposite direction, into which the wave spread.



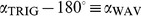
(1b)Strict *linearity* in wave propagation would support that the participation of the *focus bee* is triggered from the side where the wave came from, but not that the trigger process is launched from the opposite direction.

**Figure 5:**
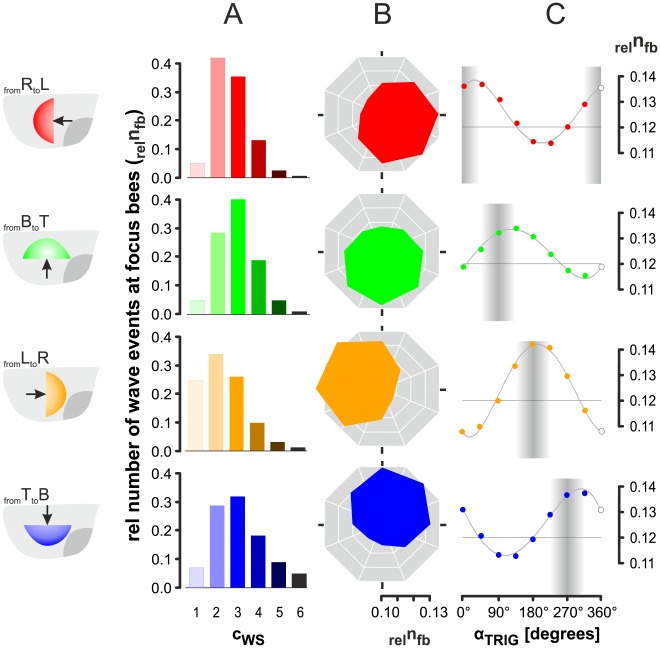
Correspondence of trigger direction and wave direction in individual *focus bees* addressing *linearity* of wave spreading in shimmering. The ordinates show the rate of wave incidents assessed at individual *focus bees* (_rel_n_fb_) and normalized for the maximum number of incidents per wave direction (_rel_n_fb_ = 1 for MAX n_fb _[dir_WAV_]). (A) Distribution of wave incidents regarding the four wave directions (dir_WAV_ = 1–4). The *focus bees* were distinguished according to their individual wave strength levels (c_ws_ = 1–6) in different colours; for evaluation, only those *focus bees* were selected that had been triggered by an immediate neighbour in the *near neighbourhood* (radius_Nh_<40 mm); *red*, from Right to Left (_from_R_to_L), n = 2825 wave incidents; *green*, from Bottom to Top (_from_B_to_T), n = 2908; *orange*, from Left to Right (_from_L_to_R), n = 2433; *blue*, from Top to Bottom (_from_T_to_B), n = 1256. (B-C) distributions of wave events regarding the *trigger directions* of individual *focus bees* (dir_TRIG_ = 1–8; for definition, see Table 1). Angular sector lines in (B) and abscissa in (C) show the *trigger angle* α_TRIG_. (C) The curves show the regression polynomials (for specification see Table 2); the grey vertical bars refer to the main wave directions coded by the median as α_WAV_ (dark grey) and the tolerances of ± 45° (bright grey).



In a first step of analysis, the *focus bees* were screened for their participation in shimmering. In all four data sets (Fig. 5A) the normalized counts _rel_n_fb_ were Gauss-distributed around mid-level strengths (c_ws_ = 2–4); the reference (_rel_n_fb_ = 1.00).

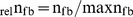
(2a)was the total number of wave incidents at a given *wave direction*. We then pooled the data of all *wave strength* levels (c_ws_ = 1–6), and sorted them according to their *trigger direction* (dir_TRIG_ = 1-8; Table 1). The data sets showed (Fig. 5B) oval-shaped angular distributions with clearly positioned minima and maxima (Fig. 5C). Furthermore, the maxima referred to those trigger angles (α_TRIG_) that conformed to the direction from where the wave came (equation 1a), while the minima were displayed at those trigger angles that conformed to the angle where the wave spread to (Equation 1b). Both results were replicated with four independent data sets, and reliably support the *directed-trigger* hypothesis which addresses the *linearity* principle in spreading (for test statistics see below).

However, the results of Fig. 5 also demonstrate that the propagation process is affected by a strikingly high level of fuzziness, unpredictability and *non-linearity*. In detail, the normalized counts _rel_n_fb_ correlated along the trigger angle with the polynomial regression curves revealing maxima of 0.152±0.004 and a distance between minima and maxima of 0.055±0.004 (n = 4 data sets of dir_WAV_; further specifications of the polynomials of Fig. 5C, see Table S1). An average base quantity of *focus bees* (0.097±0.002; n = 4 | dir_WAV_ = 1–4) was observed in each of the eight angular sectors of trigger direction. This means that for a socket of nearly 80% of *focus bees*, of a total of 100% per behavioural condition (n = 24 | c_ws_ = 1–6; dir_WAV_ = 1–4), the trigger angles did not correlate with the main wave direction.

Here, the question arises, to which extent the polynomial regression curves (Fig. 5) may then signal *linearity* in the propagation of shimmering waves. We confirmed this by a simple test considering the counts of those angular sectors (*n_s_*) which coincide in wave and trigger direction in the four data sets (dir_WAV_ = 1–4). *Linearity* would here be obvious if the _rel_n_fb_-values above median level (see ordinate values in Fig. 5) referred to the sectors of wave direction (*s*_WAV_ ≡ s_TRIG_), proving coincidence of both directional paradigms (dir_WAV_ ≡ dir_TRIG_) in the shimmering process. To test this, we expanded the critical directional window by α_WAVnew_ = α_WAVorig_ ± 45° (which increased the numbers of critical sectors in the comparison by the factor of 3). This is legitimate because the manual selection of wave direction had a similar level of tolerance (see Methods). Our results proved *linearity* because both quantities of *n_s _*.
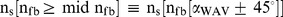
(2b)coincided here at 100% (Fig.5C). In other words, the majority of *focus bees* were triggered from the direction where the wave came from
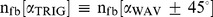
(2c)and the minority of the focus bees were triggered from the direction where the wave was spreading to



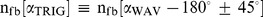
(2d)In a next step, the level of *linearity* in wave propagation was estimated in two ways (A,B). For *estimate_A_*, we integrated the percentages of participations above the fuzziness level (which was defined by the minima of the polynomials; Fig. 5C). *Estimate_A_* referred to 20.99±2.38% (n = 4 data sets of dir_WAV_) of the total of identified wave incidents and signifies the overall variability in directedness in spreading. For *estimate_B_*, we summed the percentages of participations only above the median level of _rel_n_fb_ = 0.1253±0.0015 (n = 4 data sets of dir_WAV_; equation 2e; Fig. 5C).
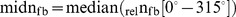
(2e)


This *estimate_B_* was 6.15±1.05% (n = 4) of identified wave incidents and included only the excess quantities of wave incidents, which were primarily responsible for the directedness of shimmering waves.

Summarizing, the data strongly support the *directed-trigger* hypothesis. However, the rates of *focus bees* conforming to equation 1a and considered by *estimate_B_* were only 6.15% of all identified wave incidents. Therefore, the principle of directedness in spreading was extremely outnumbered by the complementary part of 93.85% of *focus bees* which were triggered from random directions, independently of the main propagation direction of the waves.

### Testing *Continuity* of Wave Propagation

**Figure 6:**
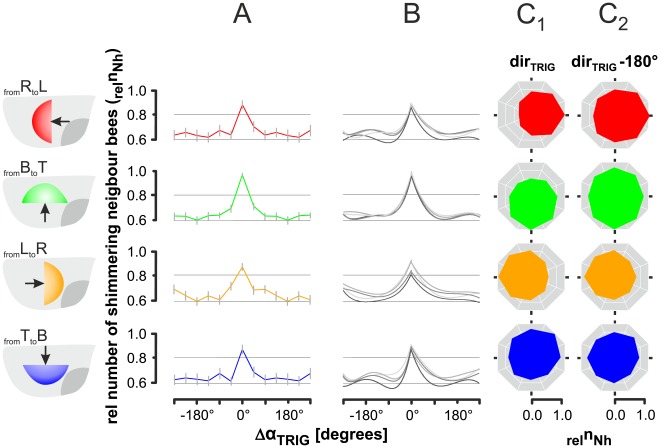
Proof of *linearity* and *continuity* in wave spreading of shimmering as documented by neighbour-bee related analysis. (A) Arithmetic means (curves) and SE (vertical bars) of the relative numbers of shimmering neighbours (_rel_n_Nh_) in the *far neighbourhood* (radius_Nh_<100 mm) around the *focus bees* regarding to the wave strength levels c_ws_ = 1-5; abscissa: Δα_TRIG | Nh_, the deviation from the trigger angle in degrees at which the *focus bees* were activated to participate in shimmering, therefore Δα_TRIG | Nh_ = 0 refers to the trigger angle (trigger direction). Four data sets representing different spreading directions of shimmering waves (for colour codes, see Fig. 5): _from_R_to_L, n_nb_ = 138190 neighbours; _from_B_to_T, 105356; _from_L_to_R, 159417; _from_T_to_B, 93199; (B) arithmetic means (curves) of the relative numbers of shimmering neighbours in the *far neighbourhood* (_rel_n_Nh_), considering four classes of wave strength levels were considered: darkest grey lines, c_ws_ = 4-5; brightest grey lines, c_ws_ = 1–2. The curves show the respective regression functions of the means (see Table S2); SEs are not shown. (C) Angular diagrams of relative numbers of shimmering neighbours (_rel_n_Nh_ ) of only those *focus bees* selected for mid-level wave strength activation (c_ws_ = 3): _from_R_to_L, n_nb_ = 4652 neighbours; _from_B_to_T, 4458; _from_L_to_R, 7182; _from_T_to_B, 2295; background sectors give the trigger angles α_TRIG_ of the *focus bees* (C_1_) *focus bees* which were triggered in the direction where the wave had come from (equation 1a); (C_2_) *focus bees* which were triggered in the direction opposite to that under C_1_ (equation 1b).



We used the sample size of the *far neighbourhood* (radius_Nh_<100 mm) of *focus bees* to characterise *continuity* in propagation by the circular distribution of nest mates which had been active in shimmering before the *focus bee* herself started to participate (Fig. 3I_2_) addressing the transmittance of information over a distance that was greater than that between immediate neighbours. The *active-neighbours* hypothesis is proved in Fig. 6 which displays the respective results of normalized data of four independent tests (dir_WAV_ = 1-4). The relative numbers of active neighbours (_rel_n_Nh_) in the *far neighbourhood.*

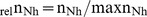
(3a)for Nh<100 mm with max _rel_n_Nh_(c_ BEHAV_) = 1.0



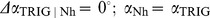
(3b)

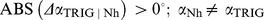
(3c)


For a better survey on the intrinsic data structure, we offer three subsets of diagrams: Fig. 6A refers to the data pool for the wave strength levels c_ws_ = 1-5, Fig. 6B distinguishes between different groups of wave strength levels (for details, see Table S2), and the circular diagrams in Fig. 6C exemplify only data for the specific wave strength level c_ws_ = 3.

In total, 48 sectors of 20 behavioural conditions (c_ws_ = 1-5, dir_WAV_ = 1–4) were evaluated. In all the four data sets the numbers of active neighbours were found to peak in the trigger directions (Δα_TRIG | Nh_ = 0°). This means that the *focus bees* were triggered exactly (P<0.001; t-test) from those directions where most of the neighbours had been active immediately before. The maxima of the graphs (Fig. 6A) increased above a level of _rel_n_Nh_ = 0.80, which corresponded to the number of n_Nh_ = 16.63±0.42 (mean ± SE) active neighbours per *focus bee*. For the angular sectors adjacent to the peaks, the graphs dropped down to _rel_n_Nh_ = 0.60, which corresponded to 11.81±0.087 neighbours per *focus bee* and 71.97% of the maximal numbers of active neighbours. The complimentary value of 28.03%.
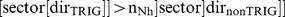
(3d)gives a usable estimate of the excess rate of neighbours found in the trigger directions of the *focus bees*. With the reference of the trigger angle of active neighbours (equation 3b) this result delivers an estimate of *continuity* in wave propagation which was fourfold higher than the *linearity* aspect detected in the context of *trigger direction* and *near neighbourhood* (Fig. 5C).

The curves of the normalized _rel_n_Nh_ values were robust regarding wave strength (Fig. 6B). Interestingly, the preferences for the wave directions were remarkably dominant (Fig. 6A-C) which is exemplified in Fig. 6C for *focus bees* acting at mid-level wave strengths (c_ws_ = 3). Here, the directional dependencies of the numbers of actively shimmering neighbours are displayed under two different conditions for the four regimes of wave directions (dir_WAV_ = 1-4): In the left column, *focus bees* were selected that corresponded in trigger direction and wave direction (according to equation 1a). The angular graphs are oval-shaped with significant (P<0.001; chi square test) preference for the wave direction. The values differed between max and min values by Δ_rel_n_Nh_ = 33%. In the right columns, the samples refer to *focus bees* that were triggered from the direction opposite to the wave direction (according to equation 1b). These wave incidents showed a much weaker but still significant (P<0.001; chi square test; Δ_rel_n_Nh_ = 22.65%) preference for the wave direction.

### Testing *Graduality* in Propagation

There are several ways how surface bees may participate in a shimmering wave. It may happen (a) as an all-or-none decision, to participate, for example, at full *wave strength*, if the surrounding activity had exceeded a certain threshold level. The circumstances of participation may be even more complex, if it were true that (b) a single *focus bee* has the freedom to decide whether or not to participate, and if she does, at which strength level. Finally, (c) it can also be a matter of *graduality* whereby *focus bees* respond with variable *wave strengths* dependent on the activity level of the surrounding neighbours.

If a *focus bee* receives the signal to start abdominal flipping as a *gradual* message from her surrounding neighbours, she has, in theory, two options: first, she could match the strength of her abdominal flipping according to the *numbers* of neighbouring bees that flipped their abdomens immediately before. This aspect has already been demonstrated in two independent test sets: we showed (a) that the rate of shimmering incidents was higher if the *focus bee* was triggered in wave direction (Fig. 5B,C), and (b), that the *focus bee* must have received stronger mechanical cues from her neighbours in her *trigger direction*, because at this angle more neighbours had been active (Fig. 6). Both findings, although highly significant, referred only to a minority of *focus bees*, at a proportion of less than 10% in Fig. 5B,C or up to a quarter in Fig. 6A,B of the full number of identified wave incidents.

As a second option, a small number of very active neighbours may also elicit a strength level that could override smaller activities of a greater number of neighbours. To address this aspect, we correlated the *wave strengths* of *focus bees* with those of their neighbours in the *far neighbourhood*. To simplify this survey, we concentrated on the neighbours in only two defined directional sectors of neighbourhood: (a) on the sectors of the trigger direction (*ds*: *direct-sector* analysis), and (b) on the sector opposite to the trigger direction (*os*: *opposite-sector* analysis). We then assessed the correlations (r_ds_, r_os_) that referred to the comparison of the *wave strengths* of *focus bees* with those of their neighbours in the selected sectors. The correlations either delivered *proportional* effects, if the wave strengths of *focus bees* correlated positively with those of their selected neighbours (with k>0).
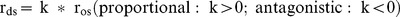
(4a)or they delivered *antagonistic* effects, if the wave strengths of *focus bees* correlated negatively (with k<0 in equation 4a).

**Figure 7:**
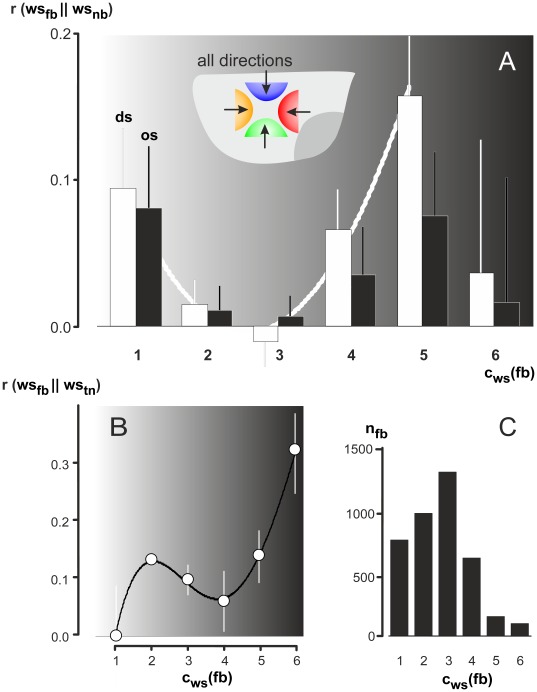
Proof of *graduality* in the wave strength between *focus bees* and their neighbours. Abscissa, wave strength levels of the *focus bee* (c_ws [fb]_ = 1–6). (A) How are the *focus bees* influenced from their neighbours in the *far neighbourhood*? Ordinate, the regression coefficients resulting from the comparison of wave strength values (_rel_XYmov) of *focus bees* (*fb*) and those of their nest mates (*nb*) in the *far neighbourhood* (r<100 mm); the data were pooled from all sets of wave directions (dir_WAV_ = 1–4); white columns refer to *direct-sector* analysis, black columns refer to *opposite-sector* analysis (for further definition, see text and equations 4a–d); the white curve refers to the distribution of *direct-sector* data (polynomial: a_2_ = 0.0324, a_1_ = -0.1763, a_0_ = 0.2379, R^2^ = 0.975; n = 5 | c_ws [fb]_ = 1–5). (B) How are *focus bees* influenced from their immediate triggering neighbours? Ordinate, regression coefficients resulting from the comparison of _rel_XYmov values of *focus bees* (*fb*) and those of their triggering neighbours (*tn*) in the *near neighbourhood* (r<40 mm), considering all wave incidences separately per wave direction (dir_WAV_ = 1 to 4); polynomial: a_4_ = −0.0037, a_3_ = 0.0668, a_2_ = −0.3926, a_1_ = 0.896, a_0_ = 0,5691, R^2^ = 0.9994; n = 6 | c_ws [fb]_ = 1–6); means (full circles) and SE (bars). (C) Numbers of wave incidences selected for B.

In detail, we pooled the data of identified *focus bees* and their neighbours under 184 test conditions (n_fb_ = 10911; n_nb_ = 496162; c_BEHAV_ = 184; dir_TRIG_ = 1–8; dir_WAV_ = 1–4; c_ws_ = 1–6) and received positive correlations under both (*ds, os*) conditions (r_ds_ = 0.0594±0.0174; r_os_ = 0.0381±0.0175; means ± SE; n_r_ = c_BEHAV_), but the regression coefficient was only slightly higher for the direct-sector analysis (P = 0.1944, t-test). In a next step, we assessed the regression data separately for each wave strength level (Fig. 7A,B; c_ws_ = 1–6) and achieved significantly positive values under weak and higher strength levels (e.g. for c_ws_ = 5: r_ds_ = 0.1600±0.0426) which also delivered significant differences between *ds-* and *os-*analysis (P = 0.0017; t-test; c_BEHAV_ = 24: dir_TRIG_ = 1-8, dir_WAV_ = 1–4; n_fb_ = 1 203; n_nb_ = 25084), in particular for the strong participation in shimmering (c_ws_ = 5). These results document that *focus bees* behave in a *gradual* and thus, *directed* manner if they responded at high *wave strengths* to the mechanical cues of those active neighbours from the trigger directions in the *far neighbourhood.* Similar responses are documented if the mechanical cues of the triggering neighbours were considered in the *near neighbourhood* (Fig. 7B). Here the correlation between the _rel_XYmov values of the *focus bees* and their triggering neighbours were also higher at higher wave strengths, and there was also a sink of regression values at mid-strength-levels. However, at threshold conditions (c_ws_ = 1) the correlations were extremely low.

Summarizing, the overall *graduality* in wave propagation displayed positive correlations between shimmering activities of *focus bees* and those of their neighbours. However, the overall achieved value with r_ds_<0.06 can be used to estimate the occurrence of *graduality* in agents participating in shimmering. The coefficient of determination R^2^ = r_ds_^2^ is here a measure of the goodness of the fit. Therefore, only 0.36% of agents participating in shimmering are controlled by the principle of *graduality*. If the *wave strength* levels were considered separately, the results proved *graduality* (Fig. 7A; equations 4a) in particular for *focus bees* acting more forcefully (c_ws_ = 5). Here, the regression coefficients achieved values of r_ds_ = 0.16 (R^2^ = 0.0256) representing 2.56% of the selected agents. These results also differed significantly between *ds*- and *os*-analyses. Such *polar* conditions preferentially occur at the very frontline of shimmering waves [14], [22]. Conversely, the bees at mid-level wave strength (c_ws_ = 2–3) which constituted up to 85% of all agents (Fig. 5A, Fig. 7C), were not identified as candidates responsible for *gradual* propagation.

## Discussion

### Properties of Wave Propagation in Shimmering

Shimmering behaviour is one element of the defence behaviour in Giant honeybees and has repelling properties upon potential predators [3]-[7]. When observed by naked eye, shimmering waves originate from particular nest areas around the mouth zone [10], [14] and propagate to the periphery in a Mexican-wavelike [21] way, spreading seemingly *linearly* and *continuously* across the nest, mostly with a clearly visible front line (movie S1). In this study, we investigated three paradigms of this wave propagation, which can be assigned as *linearity*, *continuity* and *graduality* (summarized in Fig. 3H-J), which affect direction, velocity and gain of the bucket-bridging processes [14] in shimmering.

We applied a set of recording techniques and analytical approaches that enabled us to investigate the propagation features of shimmering waves with four single-agent-based concepts (Fig. 3): (1) the (re-) identification of single agent bees at the nest surface and tracing them throughout the recorded sessions (Fig. 3B, [16]); (2) the detection of the participation of agent bees in shimmering, defining the starting time and the relative time scale of individual abdominal flipping episodes (termed as *wave incidents*), enabling synchronisation of wave incidents in subsequent pooling (Fig. 3C,D); (3) the measurement of the movement strength to quantify the participation in a wave (Fig. 3F_2_); and (4) the implementation of the concept of the *focus bee* as the addressee of the trigger process (see Methods), who receives directional information from the triggering neighbours in the *near neighbourhood* or from other agents in the *far neighbourhood* (Fig. 3F_3_). All directional properties assessed for a *focus bee* lastly refer to the partitioning of her neighbourhood into eight angular sectors (Fig. 3F_3,4_).

#### Linearity and continuity in propagation of shimmering

The *directed-trigger* hypothesis (Fig. 3H_1_–J_1_) assumes *linearity* in spreading and predicts that *focus bees* receive signals to join shimmering predominantly from the direction from where the wave spread. Confirming slight but reliable tendencies of directionality of the trigger process (Fig. 5B,C), repeatedly for four independent data sets of different spreading directions, the results supported the *directed-trigger hypothesis* for a small proportion of 6.15% of *focus bees*, but also documented a high level (93.75%) of non-*linearity* and unpredictability in the directedness of shimmering.

The paradigm of *continuity* addresses how the mode of wave propagation is preserved rather than how the directedness of spreading is established. For instance, a *discontinuous* process occurs in a shimmering wave locally if an agent bee fails to pass on the information received by an abdomen-flipping neighbour in the *bucket bridging* process [14]. Consequently, the spreading of information will either stop or by-pass to an alternative neighbour (Fig. 2B,C). Otherwise, *discontinuity* could also be a group-level property which is different to *bucket bridging.* For example, while the mechanoceptive nature of abdomen flipping is the main driving cue for *bucket bridging*, saltatoric events [14], [22] are operative only through external visual cues in the vicinity of the nest which affect some specific trigger bees [10]. Saltatoric processes forward the information of jumping-like arousal to neighbouring chains of agents some bee widths or bee lengths away. These target agents would then be able to generate daughter waves [22], but nonetheless, *bucket bridging* continues further on around such generator agents.

The aspect of *continuity* in bucket-bridging was assessed by proving the *active-neighbours* hypothesis. It predicts that most of the energy produced by abdominal flipping arrives at the *focus bee* from the *trigger direction* (Fig. 3J_2_). This hypothesis is based on two *focus bee*-specific definitions, (a) on the *trigger process* which refers to a single triggering neighbour selected from 4–6 agents in the *near neighbourhood*, and (b) on the sectoral majority of active neighbours selected from 20–30 agents in the *far neighbourhood*. *Continuity* refers to the proportion of neighbours and their angular positions from which the wave was propagated towards the *focus bee*. The results (Fig. 6) support the *active-neighbours* hypothesis proving *continuity* at a level of 27% of the full scale of identified wave incidents, confirmed through four independent tests with rich data sets.

#### Graduality in propagation of shimmering

A *gradual* transfer of information occurs if the level of participation of *focus bees* correlates with the shimmering activity of their neighbours (Fig. 3J_3_), i.e., information transfer was *gradual* if a *focus bee* passed on a signal with the same intensity that she had previously received. However, *gradual* information transfer may also deliver variable results. In particular, in the initial phase of the wave, agent bees are likely to transmit signals at an even higher energy level than their predecessors, driving the shimmering episode into the peaking phase, which usually occurred 200–500 ms after the start of the wave. In this phase, the shimmering process shows a *positive feedback* [11]. In the phase before the shimmering wave dies away, agents transmit the information in the *bucket bridging* transfer weaker than their predecessors, showing *negative feedback* [11]. In the course of a single shimmering episode, wave propagation may switch consecutively between g*raduality* and non-*graduality*, and between *positive* and *negative feedback*.

We tested *graduality* among two groups of surface bees with different memberships in bucket-bridging chains. We investigated (a) the transfer of information towards a *focus bee* from her nest mates in the *far neighbourhood* (Fig. 7A), and (b) the transfer of information towards a *focus bee* from her triggering neighbour in the *near neighbourhood* (Fig. 7B,C). These results confirm *graduality* and *positive feedback* in propagation for maximal 15% of the identified wave incidents, particularly at high wave strength levels. These conditions usually occur at the climax of the shimmering waves, but also at the very frontline of the waves, when strong mechanical *cues* are transmitted in the bee curtain for some hundreds of milliseconds.

#### Hallmarks of bucket bridging summarized

The main result of this paper is that during shimmering waves only a small minority of surface bees transmit information through *bucket bridging*. Table S3 summarizes the hallmarks of shimmering: Only 53.06±0.03% (n = 22 data sets, Table S3,c) of identified surface bees took part in shimmering; hence, the other half of the surface bees did hardly pass on any information. 75.72% of the subset of shimmering bees (corresponding to 40.18% of all surface bees, Table S3,d) were triggered by their immediate neighbours; 85% of the latter (34.15% of all surface bees, Table S3,e) responded at medium wave strengths (Fig. 5A). Only a much smaller proportion participated in shimmering either weakly (5%) or strongly (10%), corresponding to only 2.01 % (Table S3,f) or 4.02 % (Table S3,g) of the total of surface bees.

Regarding the principles of *bucket bridging* only a small minority of shimmering bees of 6.15% conformed to the concept of *linearity* (2.47% of all surface bees, Table S3,h), and 28.03% of the shimmering active bees (11.26% of all surface bees) conformed to the principle of *continuity.* Proportional transmission of information, as proposed by the *graduality* hypothesis was found in 0.36% of the surface bees, and 1.94% respectively 2.56% (Table S3,j,k) which showed strong (c_ws_ = 5 ) abdominal flipping (0.124% respectively 0.163% of surface bees).

The compilation of a wave also needs the coordination of single-agent activities to form group activities in form of semi-synchronized and cascadic sequences. *Refractory* processes are here decisive because they implement latency effects. Theoretically, without refractory processes, surface bees would hardly be able to produce any directed visual pattern such as a shimmering wave. The existence of a refractory phase would preclude that bees at the nest surface maintain abdomen-flipping activities at high frequency, after the wave front had already passed by. In shimmering, the *refractory phase* can be indirectly estimated by the repetition rate of waves under high-arousal conditions [17] which is 0.8 - 1.5 Hz; a typical value of 1.200±0.006 Hz (n = 66) was assessed at maximal arousal which would give a *refractory phase* of around 800 ms.

Interestingly, Giant honeybee colonies usually produce also collective motion patterns of abdomen-flipping surface bees, which are guided conversely to shimmering. It happens in a stochastic way, thus non-periodic and non-cascadic, and is called *flickering* [36], [24]. We have observed that *flickering* has a diurnal rhythm and happens particularly in the early morning hours. It is likely that a colony passes through an asynchronous phase of flickering before it is capable to produce the highly synchronized and cascading patterns of shimmering [3]-[7], [10], [14].

### Self Organization in Shimmering

Evidence is emerging that shimmering possesses a series of properties which categorize this behaviour as a self-organized process [11]. Two points to consider are, first, the adaptive value of the signal for external addressees. It is known [7] that shimmering can change in appearance and goal setting if a single parameter changes. For example, we found that the distance between a wasp and a Giant honeybee nest alone causes a *bifurcating* [11] situation for the attacking wasp to assess the shimmering pattern either as *defensive* or *attractive*. The second point to consider is the underlying mechanism of pattern formation. In this context, this paper gives a series of hallmark data to quantify the spatial and temporal propagation mechanisms.

Self-organised collectives such as densely clustered surface bees in Giant honeybees [11], [37] guide their actions by simple behavioural rules. One of the simplest rules that play a role in wave propagation generally [11] is that agents acquire information by monitoring their nearest neighbours. Here, this principle has been addressed for three paradigms of wave propagation in shimmering, *linearity*, *continuity* and *graduality*. The question was if and in which way these paradigms concur with the concept of self-organization [11]. For wider comparison, we consider three collective animal behaviours, fish schooling [38], bird flocking [11], [39], and fire-fly flashing [11], [40]–[42]. All these behaviours are supposedly self organized [11] and utilize neighbourhood parameters, but differ essentially in their specific design and goals.

#### Comparison of fish schooling and shimmering

Both fish schooling (and similarly bird flocking [15]) and shimmering exhibit the so-called *Trafalgar Effect* [11], [43] by which special group-level properties allow rapid transfer of information throughout the collective. In fish schools these properties happen as evasive manoeuvres [11], whereas shimmering behaviour in Giant honeybees is meant to deter external addressees [7]. Both fish schooling and shimmering occur as a wave of collective reactions that propagate essentially faster than the approaching predator [11] and group coordination is largely based on individuals which observe the preceding activity of neighbouring agents. Although the individuals time their own activities to coincide with the arrival from their neighbours [11], [39], the responses are semi-synchronous as a consequence of latency and thus resemble *bucket-bridging* processes [21]. In addition, both, fish schooling and shimmering have special group-level properties that allow a variety of tactics which may lead to striking changes of the collective formations. For instance, when threatened, fish schools [11] display evasive reactions known as the *flash expansion effect* (to rapidly expand the collective with radial bursts) or the *fountain effect* (to outmanoeuvre a predator by splitting into two groups). Similarly, shimmering in Giant honeybees is highly flexible in its dynamics, although shimmering bees essentially remain at the same location. The shape of the virtual motion patterns specifically depends on the movements of external threatening cues in space and time, such as scanning predatory wasps. In this case, the visual patterns “follow” the predator as a moving projection at the nest surface until the threatening object is “wiped off” from the nest area [7], [14], [35].

#### Comparison of firefly flashing and shimmering

Fireflies produce a coordinated rhythm of synchronised flashing [42], [11]. Each firefly primarily detects the flashes of immediate neighbours adjusting the timing of its own flash. The group pattern emerges as a result of multiple interactions among the individuals achieving flash synchrony in the collective [42], [11]. However, this kind of synchronisation happens without the concept of latency which would produce time lags in the reactions of followers. It is a matter of anticipation of the starting times of flashes which are expected from the immediate neighbours.

In Giant honeybees shimmering patterns also emerge as a result of multiple interactions of agents. But in contrast to fireflies, the primary goal of honeybees in shimmering is not to produce synchronised events, but a coordinated rhythm of cascades. The coordination of individual contributions is due to the principles of information transfer such as *bucket-bridging*, and can be *linear* and *non-linear*, *continuous* and *discontinuous*, *gradual* and *non-gradual*, with positive or negative feedback. Information is transferred from one neighbour to the next, which is coupled with the latency aspect. This means that the individuals, which follow others in their actions, “wait” for the “right” moment to participate in the wave until they receive a key signal from their neighbourhood. This is a principle of leader- and followership which generates a cascade of responses and evolves into wave-like patterns. It cannot achieve pure synchrony as in fire fly flashing. In fireflies the overall rhythm is determined by the collective timing and not imposed by any influence from outside the system, such as a leader or supervisor of external physical cues [11]. Conversely, shimmering waves depend on the activity of trigger cohorts generating parental and daughter waves [10].

The adaptive value of flash synchronization in fireflies is the production of a joint flash of males to attract females [42], [11] more than single flashes of individuals could achieve. High-level synchrony generally amplifies flash intensity, increases the range of coverage for a distant observer, and sharpens the advertence of females to visit the most attractive leks for mating [44], [45]. In contrast to the females-attracting flashes in fire flies, shimmering in giant honeybees deters potential predators [7]. The adaptive value lies in a semi-synchronous, cascading sequence of abdominal flipping of a significant sample of agents. The short latency of sequential events fuses into emergent visual patterns. This increases the range of coverage for a distant observer just by the virtual moving of the pattern.

#### The adaptive value of fuzziness in shimmering

The number of surface bees which expressed undirectedness in propagation, in the sense of *nonlinearity*, *discontinuity* and *nongraduality* exceeded 90% of the identified wave incidents (see hallmarks of *bucket brid*ging). Here, the question arises why shimmering waves have such a high level of fuzziness regarding their propagation properties. In social networks such as the bee curtain of Giant honeybees a series of reasons may cause fuzziness in propagation of information: (a) Refractory processes [17], [46] prohibit participation in shimmering immediately after wave incidents. (b) Individual surface bees possess different activation levels for abdominal flipping (Fig. 5A), which establishes a bias in the responsiveness to sense signals from the neighbours and to deliver responses towards others. (c) Lastly, Giant honeybees regularly undergo different group-level properties such as periodic mass flight activity or quiescence [25], or workloads such as the membership to the mouth zone or to quiescent areas (Fig. 1).

We propose that a high level of fuzziness in shimmering is required for rising to the challenging behaviour of scanning wasps. If this predator is to be repelled by visual patterns through shimmering, the bees have to respond to rapidly changing constellations. To follow the wasp, which usually changes the flight direction abruptly within a fraction of a second [16], [35], [24], shimmering needs an adaptive capacity of surface bees to re-direct, refresh and repeat wave generation and propagate adequate collective responses. It is known that saltatoric processes [7], [14], [35] would accelerate a wave beyond the basic capacities of *bucket bridging* [22]. Although surface bees are generally disposed to receive information via mechanical [16] or visual [14] cues, we assume that only some individuals, the *subordinate generator* bees, are able to effectively integrate both types of information [22]. These jumping-like abilities of those bees in combination with the high level of fuzziness in shimmering may provide the required speed of re-orienting and propagation of an ongoing wave. Thus, fuzziness in wave propagation may be adaptive for a more flexible propagation of visually bound signals enabling the bees to respond to rapidly changing threats.

## Supporting Information

Table S1 Equations of the polynomial regressions in Fig. 5C.
(DOC)

Table S2 Specifications of the polynomials in Fig. 6B.
(DOC)

Table S3 Summarization of numbers referring to group memberships in *bucket bridging* in Giant honeybee shimmering.
(DOC)

Movie S1 Experimental Giant honeybee (*Apis dorsata*) nest in Sauraha (Chitwan, Nepal). The computer-controlled cable-car device on the bottom right hand side moved a dummy wasp (L×W×H: 40×15×15 mm) at constant velocity and direction. The dummy wasp provoked shimmering waves which spread in a complex, repetitive pattern. The nest was situated 1 m in front of, and parallel to the back wall. The black-and-white marker with double circles on the wall in the back is 6 mm in diameter. Note the mouth zone on the bottom right hand side of the nest. The waves were generated on the left side of the mouth zone. A LED was installed on the left bottom side for triggering additional cameras and sensors. The film was recorded by a high definition camera (Panasonic DVCPRO HD) at 50 Hz.
(MOV)

Movie S2 This movie shows the same film session as displayed in movie 1, but recorded with one of the high resolution black and white cameras at 60 Hz and from another viewing angle.
(MOV)

Movie S3 This movie shows the same film session as displayed in movie S2. A single surface bee, identified as *focus bee* 20 (red closed circle marking the thorax) was selected for measurement of the movement strength _rel_XYmov. The red dot in the lower graph marks the relative observation time, and the _rel_XYmov values show the motion of the *focus bees*. Time zero of the wave incident of the *focus bee* was defined one frame before the onset of movement activity. Two wave incidents are displayed in this session. Note that there are damped oscillations due to the mechanical resonance effect of the bee curtain. The recording frequency was 60 Hz.
(MOV)
