# Supplementary material for: How to Join a Wave: Decision-Making Processes in Shimmering Behavior of Giant Honeybees (Apis dorsata)
Source: PLoS One. 2012 May 8;7(5):e36736. doi: 10.1371/journal.pone.0036736 (PMC3359778; doi:10.1371/journal.pone.0036736)
Supplement: Table S1 — Equations of the polynomial regressions in Fig. 5C. (DOC) [file pone.0036736.s001.doc]

**Table S1**. Equations of the polynomial regressions in Fig. 5C.

| **dir_WAV_** | **α_WAV_** | **c_WS_** | **a_0_** | **a_1_** | **a_2_** | **a_3_** | **a_4_** | **R²** | **n** |
| --- | --- | --- | --- | --- | --- | --- | --- | --- | --- |
| _from_R_to_L | 0° | 1-6 | 0.1374 | 0.0011 | 2xE-05 | 8xE-08 | 1xE-10 | 0,9154 | 4682 |
| _from_B_to_T | 90° | 1-6 | 0.1278 | 0.0004 | -3xE-06 | -1xE-09 | 2xE-11 | 0,9591 | 4488 |
| _from_L_to_R | 180° | 1-6 | 0.0975 | -0.0003 | 1xE-05 | -5xE-08 | 6xE-10 | 0,9143 | 3836 |
| _from_T_to_B | 270° | 1-6 | 0.1274 | -0.0007 | 6xE-06 | -2xE-08 | 3xE-11 | 0,7917 | 1543 |
|  |  |  |  |  |  |  |  |  | **14549** |
