# Supplementary material for: How to Join a Wave: Decision-Making Processes in Shimmering Behavior of Giant Honeybees (Apis dorsata)
Source: PLoS One. 2012 May 8;7(5):e36736. doi: 10.1371/journal.pone.0036736 (PMC3359778; doi:10.1371/journal.pone.0036736)
Supplement: Table S2 — Specifications of the polynomials in Fig. 6B. (DOC) [file pone.0036736.s002.doc]

**Table S2**. Specifications of the polynomials in Fig. 6B where the darkest grey lines refer to c_ws_= 4-5, and the brightest grey lines refer to c_ws_= 1-2.

| **dir_WAV_** | **α_WAV_** |  | **c_WS_** | **a_0_** | **a_1_** | **a_2_** | **a_3_** | **a_4_** | **R²** |
| --- | --- | --- | --- | --- | --- | --- | --- | --- | --- |
| _from_R_to_L | 0° |  |  |  |  |  |  |  |  |
|  |  | neg | 1-2 | 0.8323 | 0.0067 | 8.00E-05 | 4.00E-07 | 6.00E-10 | 0.9329 |
|  |  |  | 2-3 | 0.8273 | 0.0075 | 9.00E-05 | 4.00E-07 | 7.00E-10 | 0.9179 |
|  |  |  | 3-4 | 0.8308 | 0.0078 | 9.00E-05 | 4.00E-07 | 6.00E-10 | 0.8815 |
|  |  |  | 4-5 | 0.8088 | 0.007 | 7.00E-05 | 2.00E-07 | 3.00E-10 | 0.9363 |
|  |  | pos | 1-2 | 0.8388 | -0.003 | 1.00E-05 | -1.00E-08 |  | 0.9875 |
|  |  |  | 2-3 | 0.8358 | -0.003 | 1.00E-05 | -2.00E-08 |  | 0.9787 |
|  |  |  | 3-4 | 0.835 | -0.004 | 2.00E-05 | -3.00E-08 |  | 0.9437 |
|  |  |  | 4-5 | 0.8048 | -0.004 | 3.00E-05 | -5.00E-08 |  | 0.8954 |
| _from_B_to_T | 90° |  |  |  |  |  |  |  |  |
|  |  | neg | 1-2 | 0.9816 | 0.0078 | 6.00E-05 | 2.00E-07 | 2.00E-10 | 0.9950 |
|  |  |  | 2-3 | 0.9792 | 0.0077 | 6.00E-05 | 2.00E-07 | 3.00E-10 | 0.9957 |
|  |  |  | 3-4 | 0.9846 | 0.0098 | 0.0001 | 4.00E-07 | 7.00E-10 | 0.9759 |
|  |  |  | 4-5 | 0.9539 | 0.0088 | 9.00E-05 | 4.00E-07 | 6.00E-10 | 0.9935 |
|  |  | pos | 1-2 | 0.9692 | -0.006 | 4.00E-05 | -7.00E-08 |  | 0.9600 |
|  |  |  | 2-3 | 0.9712 | -0.006 | 4.00E-05 | -7.00E-08 |  | 0.9819 |
|  |  |  | 3-4 | 0.9782 | -0.007 | 4.00E-05 | -8.00E-08 |  | 0.9770 |
|  |  |  | 4-5 | 0.945 | -0.007 | 4.00E-05 | -7.00E-08 |  | 0.9798 |
| _from_L_to_R | 180° |  |  |  |  |  |  |  |  |
|  |  | neg | 1-2 | 0.8757 | 0.0044 | 3.00E-05 | 1.00E-07 | 2.00E-10 | 0.9557 |
|  |  |  | 2-3 | 0.8714 | 0.0048 | 3.00E-05 | 1.00E-07 | 1.00E-10 | 0.9872 |
|  |  |  | 3-4 | 0.8016 | 0.0034 | 2.00E-05 | 3.00E-08 | 5.00E-12 | 0.9784 |
|  |  |  | 4-5 | 0.7771 | 0.0041 | 4.00E-05 | 2.00E-07 | 3.00E-10 | 0.9199 |
|  |  | pos | 1-2 | 0.8606 | 0.004 | 2.00E-05 | -3.00E-08 |  | 0.8740 |
|  |  |  | 2-3 | 0.8587 | 0.0032 | 2.00E-05 | -3.00E-08 |  | 0.9272 |
|  |  |  | 3-4 | 0.791 | 0.0026 | 1.00E-05 | -3.00E-08 |  | 0.9583 |
|  |  |  | 4-5 | 0.766 | 0.0028 | 1.00E-05 | -3.00E-08 |  | 0.8894 |
| _from_T_to_B | 270° |  |  |  |  |  |  |  |  |
|  |  | neg | 1-2 | 0.8235 | 0.0062 | 7.00E-05 | 3.00E-07 | 6.00E-10 | 0.8961 |
|  |  |  | 2-3 | 0.8325 | 0.0086 | 0.0001 | 6.00E-07 | 1.00E-09 | 0.9226 |
|  |  |  | 3-4 | 0.808 | 0.0082 | 0.0001 | 5.00E-07 | 8.00E-10 | 0.8781 |
|  |  |  | 4-5 | 0.7982 | 0.0088 | 1.00E-04 | 4.00E-07 | 6.00E-10 | 0.9118 |
|  |  | pos | 1-2 | 0.8274 | 0.003 | 1.00E-05 | -6.00E-09 |  | 0.9894 |
|  |  |  | 2-3 | 0.8417 | 0.0034 | 2.00E-05 | -2.00E-08 |  | 0.9737 |
|  |  |  | 3-4 | 0.8157 | 0.0032 | 1.00E-05 | -2.00E-08 |  | 0.9701 |
|  |  |  | 4-5 | 0.7957 | 0.0049 | 3.00E-05 | -6.00E-08 |  | 0.9197 |
