# Supplementary material for: How to Join a Wave: Decision-Making Processes in Shimmering Behavior of Giant Honeybees (Apis dorsata)
Source: PLoS One. 2012 May 8;7(5):e36736. doi: 10.1371/journal.pone.0036736 (PMC3359778; doi:10.1371/journal.pone.0036736)
Supplement: Table S3 — Summarization of numbers referring to group memberships in bucket bridging in Giant honeybee shimmering. (DOC) [file pone.0036736.s003.doc]

**Table S3.** Summarization of numbers referring to group memberships in bucket bridging in Giant honeybee shimmering

|  | **Membership of surface bees** |  | **numbers** | **References** |
| --- | --- | --- | --- | --- |
| [a] | Number of identified agents |  | 496162 | Fig. 3B-D |
| [b] | Number of identified *focus bees* |  | 14549 | Fig. 3D |
|  |  | **Partial percentages** | |  |
|  |  |  | of surface bees (n=22 data sets) |  |
| [c] | Rel number of identified surface bees which take part in shimmering |  | 53.06% ± 3.05 | Fig. 3E-G |
| [d] | -- Of those were triggered by immediate neighbours | 75.72% of [c] ±2,90 | = 40.18% | Fig. 3H, 4 |
| [e] | -- -- Of those responded at medium wave strengths (c_ws_= 2-4) | 85% of [d] | = 34.15% | Fig. 3H, 5A |
| [f] | -- -- Of those participated at weaker strength (c_ws_= 1) | 5% of [d] | = 2.01% | Fig. 3H, 5A |
| [g] | -- -- Of those participated at biggest strength (c_ws_≥ 6) | 10% of [d] | = 4.02% | Fig. 3H, 5A |
|  | **LINEARITY** |  |  |  |
| [h] | Rel number of identified *focus bees* conforming to the concept of *linearity* | 6.15% of [d] | = 2.47% | Fig. 3H, 5 |
|  | **CONTINUITY** |  |  |  |
| [i] | Rel number of identified *focus bees* conforming to the concept of *continuity* | 28.03% of [d] | = 11.26% | Fig. 3H, 6 |
|  | **GRADUALITY** |  |  |  |
|  | Rel number of identified *focus bees* conforming to the concept of *graduality* |  |  |  |
| [j] | -- Number of focus bees which show strong abdominal flipping (c_ws_= 5) | 15.86% of [d] | = 6.37% | Fig. 3H, 7BC |
| [k] | -- -- Of which show *graduality* (between *focus bees* and their *trigger neighbours*) | 1.94% (*R^2^*=0.0194) | = 0.124% | Fig. 3H, 7A |
| [l] | -- -- Of which show *graduality* (between *focus bees* and their neighbours in the *far neighbourhood*) | 2.56% (*R^2^*=0.0256) | = 0.163% | Fig. 3H, 7A |
